# Prediction of novel long non-coding RNAs based on RNA-Seq data of mouse Klf1 knockout study

**DOI:** 10.1186/1471-2105-13-331

**Published:** 2012-12-13

**Authors:** Lei Sun, Zhihua Zhang, Timothy L Bailey, Andrew C Perkins, Michael R Tallack, Zhao Xu, Hui Liu

**Affiliations:** 1 School of Information and Electrical Engineering, China University of Mining and Technology, Xuzhou, 221008, JiangSu, PR China; 2 Center for Computational Biology, and Laboratory of Disease Genomics and Personalized Medicine, Beijing Institute of Genomics, Chinese Academy of Sciences, No.7 Beitucheng West Road, Chaoyang District, Beijing, 100029, PR China; 3 Institute for Molecular Bioscience, The University of Queensland, Brisbane, 4072, Queensland, Australia; 4 Mater Medical Research Institute, Mater Hospital, Brisbane, 4101, Queensland, Australia

## Abstract

**Background:**

Study on long non-coding RNAs (lncRNAs) has been promoted by high-throughput RNA sequencing (RNA-Seq). However, it is still not trivial to identify lncRNAs from the RNA-Seq data and it remains a challenge to uncover their functions.

**Results:**

We present a computational pipeline for detecting novel lncRNAs from the RNA-Seq data. First, the genome-guided transcriptome reconstruction is used to generate initially assembled transcripts. The possible partial transcripts and artefacts are filtered according to the quantified expression level. After that, novel lncRNAs are detected by further filtering known transcripts and those with high protein coding potential, using a newly developed program called lncRScan. We applied our pipeline to a mouse *Klf1* knockout dataset, and discussed the plausible functions of the novel lncRNAs we detected by differential expression analysis. We identified 308 novel lncRNA candidates, which have shorter transcript length, fewer exons, shorter putative open reading frame, compared with known protein-coding transcripts. Of the lncRNAs, 52 large intergenic ncRNAs (lincRNAs) show lower expression level than the protein-coding ones and 13 lncRNAs represent significant differential expression between the wild-type and *Klf1* knockout conditions.

**Conclusions:**

Our method can predict a set of novel lncRNAs from the RNA-Seq data. Some of the lncRNAs are showed differentially expressed between the wild-type and *Klf1* knockout strains, suggested that those novel lncRNAs can be given high priority in further functional studies.

## Background

The category of long non-coding RNAs (lncRNAs) is composed of non-coding RNAs (ncRNAs) with long transcript length (> 200 nucleotides) [1]. The lncRNAs may carry out a variety of functions, e.g. scaffolding multiple proteins to form a complex, and regulating gene expression [2-11], however, most lncRNAs’ functions remain to be specified. During the past decade, a growing number of newly detected lncRNAs have been reported thanks to the development of relevant biotechnology and computational methods [4,12-16]. Early tiling microarrays were used to detect the lncRNAs in the mammalian transcriptome [4,5], however, they could not detect precise gene structures and exon linkages of the lncRNAs [14]. Subsequently, this problem was tackled by high-throughput RNA sequencing (RNA-Seq), which presented its advantage of revealing the whole transcriptome [17], including detailed gene structures and expression levels. So far, the RNA-Seq has been the major biotechnology for lncRNA study [13]. For example, by using RNA-Seq, Guttman et al. [14] obtained detailed information of over a thousand large intergenic ncRNAs (lincRNAs) in three mouse cell types [14].

However, studying lncRNAs based on RNA-Seq encounters several technical problems. First, the assembled transcriptome may include partial transcripts and artefacts caused by RNA-Seq problems, such as low sequencing depth, sequencing biases [18] and short read alignment errors [19]. For lowly expressed transcripts, the sequencing biases may introduce undesired gaps in the assembly, resulting in partially assembled transcripts [20], which may be mistakenly identified as lncRNAs. The similar mistakes could also be introduced by low sequencing depth for lowly expressed transcripts. Moreover, the incomplete and erroneous assemblies can affect downstream analysis [21,22]. Second, transcriptome reconstruction [23] based on RNA-Seq reads may produce a variety of transcripts, e.g. completely assembled transcripts, intronic RNAs [24] and antisense transcripts [16], which are classified by comparing to the known gene annotations. Thus it is not trivial to identify lncRNAs from such complex assemblies. Third, it is still difficult to distinguish the lncRNAs from the protein-coding mRNAs [1] or short peptides. A protein-coding mRNA can be defined by open reading frame (ORF) greater than 100 amino acids (aa) or 300 nucleotides (nt) [25], but this is arbitrary and incorrect [26]. Here we present a computational pipeline to address these problems.

Although thousands of lncRNAs have been identified [13,14,16], only a handful of them were functionally characterized. Given the difficulty to experimentally characterize the biological functions of the lncRNAs [7], and given the growing body of genomics and epigenomics data becoming available relevant to lncRNAs’ biological functions, it is interesting to predict lncRNAs’ functions computationally. We applied our computational method to an RNA-Seq dataset derived from a *Klf1* gene knockout study on mouse fetal liver tissue [27]. Previous studies based on the *Klf1* knockout study manifested that *Klf1* is the founding member of a family of 17 transcription factors in mammals [28]. *Klf1* knockout mice die from anemia by embryonic day 15 (E15), with severe defects in differentiation, hemoglobinization, enucleation, and membranecytoskeleton organization of red blood cells [29]. However, very little is known of the lncRNAs regulated by *Klf1* or that participate in the development of erythroid cells. Here, we recruit the differential expression analysis to explore the lncRNAs that may function in the erythropoiesis.

## Methods

### Datasets

The RNA-Seq dataset for the *Klf1* knockout experiment on mouse embryonic day 14.5 (E14.5) fetal liver tissue can be obtained from NCBI Gene Expression Omnibus (GEO) [30] database with accession number GSE33979 [27], and it includes 6 replicates (3 for wild-type and 3 for *Klf1* knockout) totalling 160 million 76-base single-end reads generated by Illumina GAIIx sequencing on polyadenylated selected (Poly-*A*^+^) RNAs. Bowtie [31] index of *Mus musculus* genome (mm9), Ensembl [32] and NCBI reference sequences (RefSeq) mouse gene annotations [33] are all available on Cufflinks’ website [34]. University of California Santa Cruz (UCSC) mouse known gene annotations [35] can be downloaded from the UCSC genome browser [36].

### Pipeline for predicting novel lncRNAs

**Figure 1:**
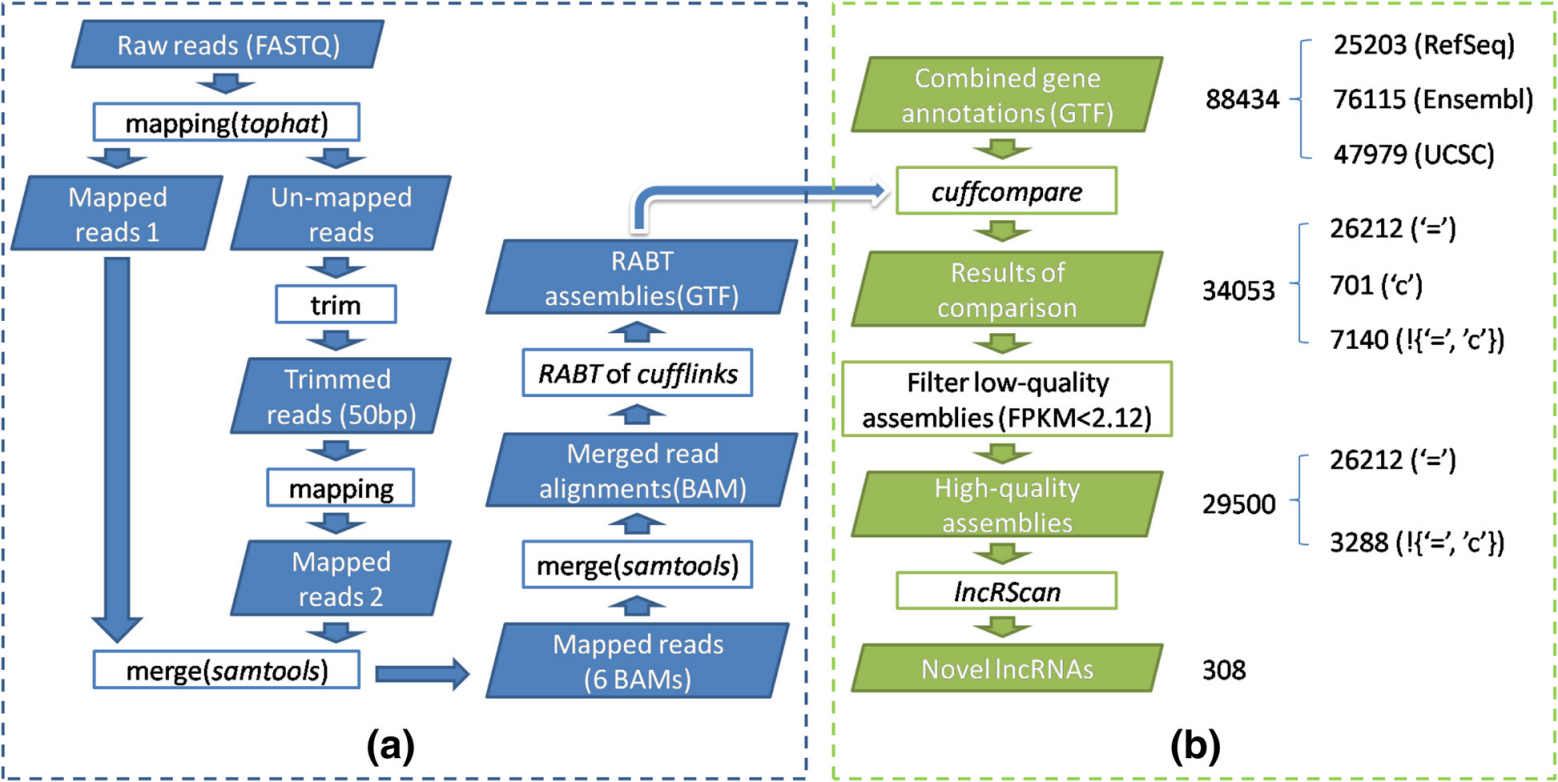
**Pipeline for predicting novel lncRNAs. (a)** Initial assembly. Raw reads are first mapped onto the reference mouse genome. The un-mapped reads are trimmed before re-mapping. Merging the read alignments of all 6 replicates is to increase the read coverage. At the assembly stage, RABT generates synthetic reads from the RefSeq gene annotation to compensate the read coverage gaps over transcripts; **(b)** Novel lncRNAs detection. The initial assemblies are categorized by cuffcompare, compared with the combined gene annotations. The low-quality transcripts are then filtered according to the optimum FPKM (2.12). The lncRScan program is performed to detect the novel lncRNAs from the remaining high-quality assemblies according to multiple criteria.

There are two parts in our pipeline for predicting novel lncRNAs from the RNA-Seq data (Figure 1).

#### Initial assembly

Initial assembly (Figure 1-a) represents a genome-guided strategy for transcriptome reconstruction [23]. The raw RNA-Seq reads were first mapped onto the mm9 genome by Tophat 2.0.3 [19]. After that, the un-mapped reads were trimmed to 50 nt before re-mapping. The final mapped reads of each replicate include two parts, namely ‘Mapped reads 1’ and ‘Mapped reads 2’. Moreover, the ‘-G’ option of Tophat together with the **G**ene **T**ransfer **F**ormat (GTF) file of the Ensembl gene annotation was used for read mapping. With the read alignments, we calculated the overlap ratio (OR) between the replicates of each condition (Additional file 1). To increase the read coverage, we merged the read alignments of all six replicates into one **B**inary version of Sequence **A**lingment/**M**ap (BAM) using Samtools 0.1.18 [37]. Then the mapped reads were assembled by Cufflinks 2.0.2 [21]. In the transcriptome assembly, we performed **R**eference **A**nnotation **B**ased **T**ranscript (RABT) assembly [38] with the RefSeq gene annotation to compensate incompletely assembled transcripts caused by read coverage gaps in the regions of RefSeq genes.

#### Novel lncRNAs detection

Novel lncRNAs detection (Figure 1-b) is aimed at detecting novel lncRNAs from the initial assemblies. Specifically, the initial assemblies were first compared to a set of combined gene annotations (See below) using cuffcompare [22]. As a result, not only the assemblies that completely match the annotations will be detected, but also the novel transcripts can be categorized into different categories according to their locations compared with the reference genes. Notably, only multi-exon transcripts were retained for the comparison and downstream processing. Then low-quality assemblies were filtered according to the optimum **F**ragments **P**er **K**ilobases of exon per **M**illion fragments mapped (FPKM) [21] threshold (2.12, see below). After that, we used a newly-developed program called lncRScan (See below) to detect novel lncRNAs.

### Combined gene annotations of RefSeq, Ensembl and UCSC mouse known genes

The cuffcompare program [22] was used to merge the RefSeq, Ensembl and UCSC mouse known genes into one set of gene annotation for comparing with the assembled transcripts.

### FPKM threshold for classifying complete and partial transcripts

Based on the merged read alignments, we conducted an experiment to evaluate the performance of FPKM in classifying complete and partial transcripts. Specifically, we first ran cufflinks on the merged read alignments with default options. Then the output assemblies with FPKM values estimated were categorized using cuffcompare, compared with the combined gene annotations. With the results, we evaluated the performance of different FPKM thresholds in classifying the complete and partial transcripts by **R**eceiver **O**perating **C**haracteristic (ROC) [39].

### Calculating optimum FPKM threshold

The optimum FPKM threshold for classifying the complete and partial transcripts were calculated by training the FPKM values estimated from the experiment above. The index of the optimum FPKM threshold can be obtained by optimizing the sensitivity and specificity in classifying the complete and partial transcripts with formula 1.

(1) $$\;i^{*}\;=\;\underset{i\in I}{\text{arg min}}\left\{\sqrt{{\left(1-\text{sensitivities}[i]\right)}^{2}+{\left(1-\text{specificities}[i]\right)}^{2}}\right\}$$

where *i*^∗^represents the index of the optimum FPKM threshold. On the right of formula 1, *sensitivitiesi* and *specificitiesi* respectively denote the *i*th *sensitivities* and *specificities*, given an index *i*. The *i* is enumerated in *I*, ranging from 1 to the size of a FPKM threshold set. Then we can get the optimum FPKM threshold using formula 2.

(2) $$t^{*}=T[i^{*}]$$

where *t*^∗ ^denotes the optimum FPKM threshold. The FPKM threshold set *T* were generated by pROC [39], given the FPKM values of the complete and partial transcripts.

### lncRScan

**Figure 2:**
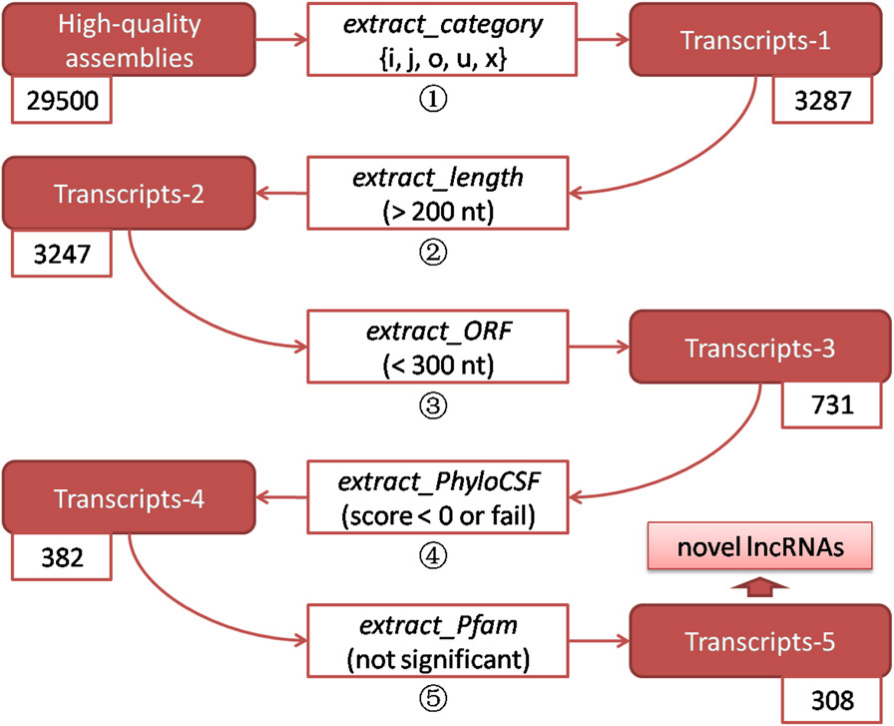
**Steps of lncRScan.** (1) ‘extract_category’ extracts five candidate categories of assemblies (Transcripts-1), including ‘i’, ‘j’, ‘o’, ‘u’ and ‘x’; (2) ‘extract_length’ is used to extract the transcripts with length > 200 nt (Transcripts-2); (3) ‘extract_ORF’ selects the transcripts with maximum putative ORF < 300 nt (Transcripts-3); (4) ‘extract_PhyloCSF’ extracts the transcripts with PhyloCSF score < 0 or test failure due to ORF < 25 aa (Transcripts-4); (5) ’extract_Pfam’ searches the remaining transcripts in the Pfam database and excludes the transcripts with significant protein domain hits. Towards the end of lncRScan, the remaining 308 transcripts (Transcripts-5) are defined as the novel lncRNAs.

To detect novel lncRNAs from a set of high-quality assemblies, a five-step program named **l**ong **n**on-**c**oding **R**NA **S**can (lncRScan) was designed (Figure 2). Step 1 ‘extract_category’ is used to extract five candidate categories of transcripts, including ‘i’, ‘j’, ‘o’, ‘u’ and ‘x’, which may contain novel lncRNAs. Specifically, the ‘i’ category may contain the lncRNAs falling entirely within an intron of known genes. And the ‘j’ category may include alternative long non-coding isoforms of known genes as they share at least one spliced site with reference transcripts. The ‘u’ category may involve the intergenic lncRNAs (lincRNAs). The ‘o’ category may contain the lncRNAs having generic exonic overlap with a known transcript while the ‘x’ transcripts also have exonic overlap with reference but on the opposite strand. Therefore, the five categories defined here may include novel lncRNAs potentially. On the other hand, all categories of transcripts extracted have not been annotated by either of RefSeq, Ensembl and UCSC known genes, so the predicted lncRNAs can be ‘novel’. Step 2 ‘extract_length’ is used to extract the transcripts having long exonic length (> 200 nt) according to the lncRNA’s definition. Step 3 ‘extract_ORF’ is set to exclude the assemblies that have long (≥ 300 nt) putative ORF. Then steps 4 and 5 are used to exclude the transcripts of protein-coding potential. In Step 4 ‘extract_PhyloCSF’, **Phylo**genetic **C**odon **S**ubstitution **F**requency (PhyloCSF) [40] is recruited to filter the transcripts of protein-coding potential from an evolutionary view. Briefly, PhyloCSF conducts a comparative genomics method for classifying protein-coding and non-coding sequences [40]. Since the sequence alignments are required for running PhyloCSF, we used Galaxy [41-43] to ‘stitch’ 29 mammalian alignments according to the input transcripts. In Step 5 ‘extract_Pfam’, the amino acid sequences of the remaining transcripts are searched in Pfam [44] (both Pfam-A and Pfam-B) for comparing to known proteins or protein domains, and the transcripts with significant domain hits are excluded.

To evaluate the performance of lncRScan in identifying lncRNAs or filtering mRNAs, we ran the steps 3-5 of lncRScan on four datasets respectively. The first dataset (D-1) contains 1615 multi-exon RefSeq ncRNAs with length > 200nt and the second one (D-2) records 1615 mRNAs randomly sampled from 26368 RefSeq mRNAs. The other two datasets (D-3 and D-4) include 3230 and 4845 mRNAs sampled from the RefSeq mRNAs respectively. The numbers of the retained and filtered transcripts through the steps 3-5 of lncRScan are summarized in Table 1. We can see that 771 (47.74%) lncRNAs of D-1 were retained after the steps 3-5. In contrast, most (99.6%-99.7%) of the mRNAs (D-2, D-3 and D-4) were filtered by the steps 3-5. The result indicates that the filters of lncRScan can dramatically reduce the number of mRNAs. Notably, the step 3 adopting the ORF threshold can filter a large proportion of mRNAs thereby alleviating the overload of PhyloCSF and Pfam calculation. However, some true lncRNAs were filtered through the pipeline, which made the final lncRNAs prediction much stringent.

**Table 1:** Numbers of retained and filtered transcripts through steps 3-5 of lncRScan

**Test data**	**extract_ORF(Step 3)**		**extract_PhyloCSF(Step 4)**		**extract_Pfam(Step 5)**	
	**Retained**	**Filtered**	**Retained**	**Filtered**	**Retained**	**Filtered**
D-1 (1615 lncRNAs)	952(58.95%)	663	813(50.34%)	139	771(47.74%)	42
D-2 (1615 mRNAs)	33(2%)	1582	12(0.74%)	21	6(0.37%)	6
D-3 (3230 mRNAs)	89(2.76%)	3141	45(1.44%)	44	10(0.31%)	35
D-4 (4845 mRNAs)	112(2.31%)	3733	50(1.03%)	62	18(0.37%)	32

In addition, lncRScan is available to the scientific community and it can be obtained by *svn checkout*http://lncrscan.googlecode.com/svn/trunk/*lncrscan-read-only*. Other details about lncRScan can be found on http://code.google.com/p/lncrscan/.

### Differential expression analysis

**Figure 3:**
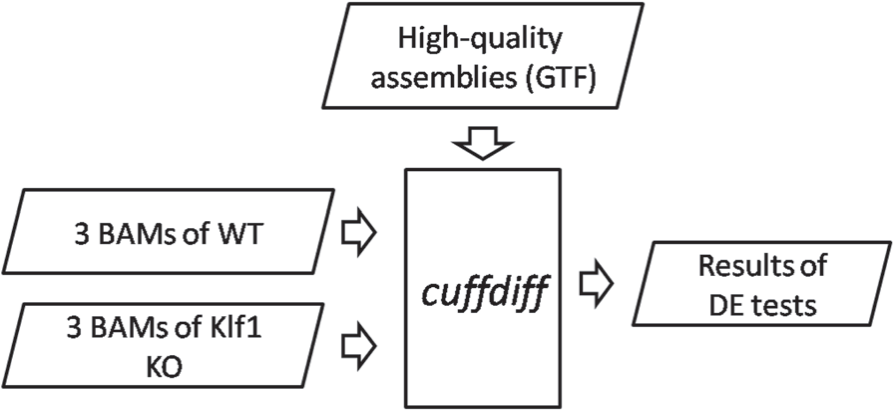
**Differential expression tests.** The cuffdiff program performs differential expression tests between the WT and *Klf1* KO samples based on the read alignments (BAM) of the six replicates and high-quality assemblies (GTF).

The cuffdiff [22] program was performed to conduct differential expression (DE) tests between the wild-type (WT) and *Klf1* knockout (*Klf1* KO) samples (Figure 3). The fold changes were calculated via $\mathrm{lo}g_{2}\frac{\text{FPKM}\;\text{WT}}{\text{FPKM}\;\text{Klf}1\text{KO}}$. A transcript will be reported DE significant if the test gives that the FDR-adjusted p-value after Benjamini-Hochberg correction [45] for multiple-testing represent statistical significant (q-value < 0.05) [46].

### Comparisons of transcript length, exon number, ORF length and expression level

The novel lncRNAs we detected were compared to 26368 RefSeq protein-coding transcripts (‘NM’ prefix) and 2843 RefSeq non-coding transcripts (‘NR’ prefix) in terms of transcript length, exon number and ORF length. Since a real ncRNA does not have an ORF, a putative ORF of the ncRNA candidate is defined by the longest consecutive codon chain of the ncRNA candidate for comparing with the protein-coding genes. Moreover, for both of the WT and *Klf1* KO conditions, we compared the quantified expression levels (FPKM) of the novel lncRNAs to that of the known protein-coding transcripts, which were extracted from the RefSeq and Ensembl gene annotations. The novel lncRNAs and protein-coding transcripts used for FPKM comparison all have enough expression levels (FPKM ≥ 2*.*12).

## Results

### Initially assembled transcripts

We started our analysis with short read mapping (Figure 1-a), and approximately 138 million reads were successfully mapped onto the mm9 genome (Table 2). With the merged alignments of six replicates, 34053 multi-exon transcripts (26212 annotated, 701 contained by annotations and 7140 novel potentially) were assembled in total, compared with 88434 transcripts of the combined gene annotations. Then we obtained the categories of the initial assemblies by comparing to the combined gene annotations (Table 3). It is notable that the initial assemblies include several categories of transcripts, e.g. transcripts that have complete match intron chain compared with known genes (‘=’ classcode) and those contained by known genes (‘c’ classcode). Of the initial assemblies, 26212 (76.97%) transcripts have been annotated by either of RefSeq, Ensembl and UCSC known genes.

**Table 2:** Read mapping summary

**Replicate**	**Raw reads**	**Un-mapped**	**Mapped**
KO_1	25153995	5713351 (22.7%)	19440644 (77.3%)
KO_2	26269828	3294901 (12.5%)	22974927 (87.5%)
KO_3	25988788	6032342 (23.2%)	19956446 (76.8%)
WT_1	20034326	2006957 (10.0%)	18027369 (90.0%)
WT_2	22221706	4486281 (20.2%)	17735425 (79.8%)
WT_3	45034903	4678496 (10.4%)	40356407 (89.6%)
total	164703546	26212328 (15.9%)	138491218 (84.1%)

**Table 3:** Categories of initial assemblies

**Class code**	**Transcript number**	**Percentage**	**Description**
=	26212	76.97%	Complete match of intron chain
c	701	2.06%	Contained by a reference transcript
j	6207	18.23%	At least one splice junction is shared with a reference transcript
i	155	0.46%	A transfrag falling entirely within a reference intron
o	187	0.55%	Generic exonic overlap with a reference transcript
u	492	1.44%	Unknown, intergenic transcript
x	98	0.29%	Exonic overlap with reference on the opposite strand
s	1	0.00%	An intron of the transfrag overlaps a reference intron on the opposite strand
total	34053	100%	Total

### Filtering low-quality assemblies with optimum FPKM threshold

**Figure 4:**
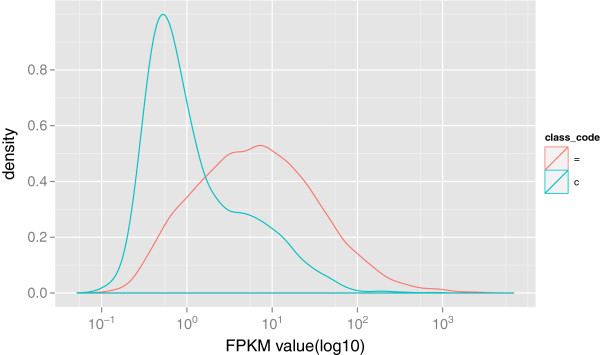
**FPKM distributions of complete and partial transcripts.** The ‘=’ classcode is originally assigned to the transcripts that have complete match intron chain with a reference transcript and they can be treated as complete transcripts while the ‘c’ classcode is attached to the transcripts contained by reference and they are defined as partial assemblies. The complete (‘=’, red curve) and partial (‘c’, blue curve) transcripts assembled from the read alignments represent distinguishable FPKM distributions from each other (∼29.67 vs ∼4.86).

**Figure 5:**
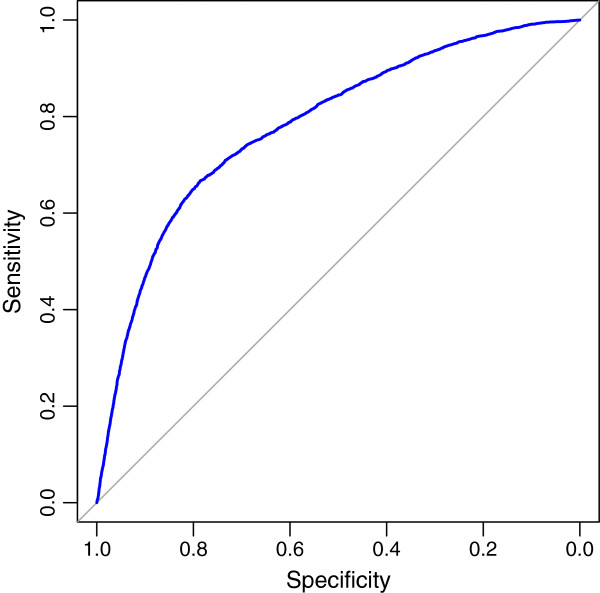
**Performance of FPKM in distinguishing between complete and partial transcripts.** An assembled transcript will be classified into the category of complete assemblies (‘=’ classcode) if its FPKM is larger than a given threshold, otherwise it will be put into the partial category (‘c’ classcode). The blue ROC curve [39] represents the performance of FPKM in classifying the complete and partial transcripts. The corresponding **A**rea **U**nder **C**urve (AOC) is 0.7825.

FPKM can unbiasedly represent quantified expression level of an assembled transcript, and it can be estimated by maximum likelihood estimation (MLE) under a statistical model of cufflinks [21], which also corrects sequencing biases [18] in the estimation. Figure 4 shows the FPKM distributions [47] of the complete (‘=’ classcode) and partial (‘c’ classcode) transcripts assembled from the experiment of FPKM threshold (See Methods) while Figure 5 shows the corresponding ROC curve. Notably, the complete transcripts represent much larger FPKM than the partial ones on average (∼29.67 vs ∼4.86, *P *< 2*.*2 × 10^−16^, Welch Two Sample t-test). According to the significant difference of FPKM distributions of complete and partial assemblies, we calculated the optimum FPKM threshold (2.12) based on our data (See Methods). We assumed that the artificial transcripts represent either similar FPKM distribution to the partial transcripts or lower FPKM than the partial ones, thus the optimum threshold can be used to filter both of the partial assemblies and artefacts from the 7140 novel assemblies.

### Identification of high-quality assemblies

We pooled a set of high-quality assemblies (Additional file 2) for downstream analysis. The high-quality assemblies consist of two categories. One category contains the 26212 initial assemblies that completely match the combined gene annotations (‘=’ classcode). The other category refers to the 3288 transcripts extracted from the 7140 novel assemblies (!{‘=’,‘c’}), which satisfy the expression criterion (FPKM ≥ 2.12).

### Novel mouse embryonic lncRNAs

**Figure 6:**
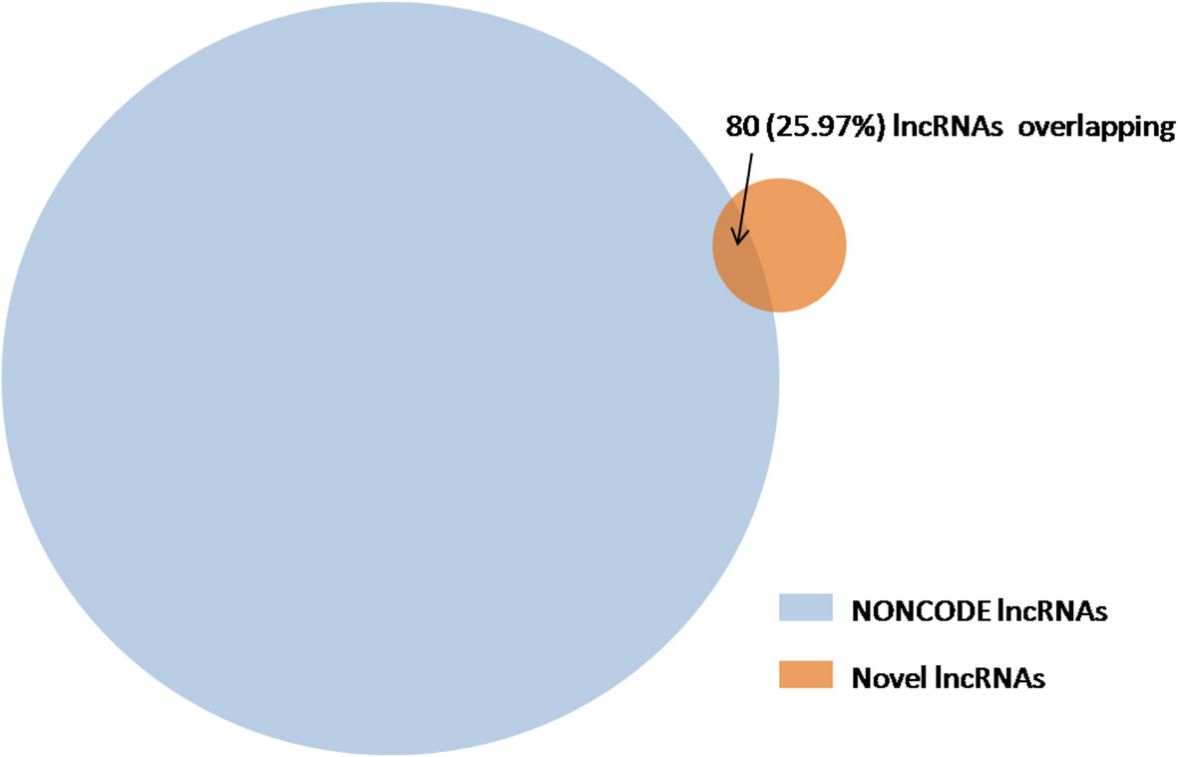
**Comparison between novel lncRNAs and NONCODE lncRNAs.** There are 36991 lncRNAs annotated by NONCODE 3.0 and 308 lncRNAs predicted by our method. Of the 80 (25.97% of our prediction) overlapped lncRNAs, 5 ones have been exactly annotated by NONCODE 3.0

We applied our newly developed lncRNAs detector lncRScan to the high-quality assemblies and detected 308 novel mouse embryonic lncRNAs (Additional file 3). The novel lncRNAs were further classified into 5 categories by comparing with the known gene annotations (Table 4). Specifically, 52 lncRNAs were assigned the ‘u’ classcode since they were located in the intergenic regions. And 26 lncRNAs with the ‘i’ classcode fall entirely within the intron of known genes. The other lncRNAs all have exon overlap with known genes. Specifically, 44 lncRNAs with the ‘o’ classcode have generic exonic overlap with known genes and 6 ‘x’ lncRNAs also have exonic overlap with known genes but on the opposite strand. The 180 lncRNAs with ‘j’ can be long non-coding isoforms of known genes. In addition, the 308 novel lncRNAs we predicted were compared with 36991 ones annotated by NONCODE 3.0 [48]. Of the 308 novel lncRNAs, 5 (1.62%) ones have the same structure as NONCODE lncRNAs (Additional file 1) and another 75 (24.35%) ones partially overlap the NONCODE lncRNAs (Figure 6). By excluding the 80 lncRNAs that overlap the NONCODE annotation, we can get a more stringent set of novel lncRNAs.

**Table 4:** Categories of novel lncRNAs

**Class code**	**Transcript number**	**Percentage**	**Description**
j	180	58.44%	At least one splice junction is shared with a reference transcript
i	26	8.44%	A transfrag falling entirely within a reference intron
o	44	14.29%	Generic exonic overlap with a reference transcript
u	52	16.88%	Unknown, intergenic transcript
x	6	1.95%	Exonic overlap with reference on the opposite strand
total	308	100%	Total

### Novel lncRNAs have shorter transcript length, fewer exons and shorter putative ORF than protein-coding transcripts

**Figure 7:**
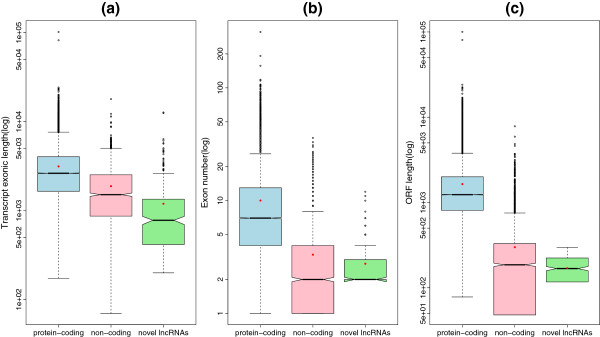
**Comparisons of transcript length, exon number and ORF length. (a)** Comparison of transcript length. The novel lncRNAs show shorter length (∼1.2kb) on average than either RefSeq protein-coding (∼3.1kb) or non-coding transcripts (∼1.9kb); **(b)** Comparison of exon number. The lncRNAs represent fewer exons (∼2.8) than the other two categories of transcripts (∼10.0 and ∼3.3, respectively) on average; **(c)** Comparison of ORF length. The novel lncRNAs show shorter putative ORF length (∼0.17kb) than either of the two RefSeq gene categories (∼1.6kb and ∼0.3kb, respectively) on average. All means are marked by red points

Previous studies in mammals have shown that lncRNAs are shorter in length and fewer in exon number than are protein-coding transcripts [13,14,16]. To determine whether the embryonic lncRNAs we detected have the same features, we compared the 308 novel lncRNAs to not only 26368 protein-coding transcripts, but also 2843 known non-coding ones, annotated by RefSeq (See Methods). As shown in Figure 7, the novel lncRNAs represent much shorter transcript length on average than either RefSeq protein-coding (∼1.2kb vs ∼3.1kb, *P *< 2*.*2×10^−16^, Welch Two Sample t-test) or non-coding transcripts (∼1.2kb vs ∼1.9kb, *P *= 6*.*027 × 10^−14^) while the lncRNAs also show fewer exons than either of the RefSeq protein-coding (∼2.8 vs ∼10.0, *P *< 2*.*2 × 10^−16^) and non-coding transcripts (∼2.8 vs ∼3.3, *P *= 5*.*096 × 10^−8^), agreed with a previous report [13]. In addition, we also compared the putative ORF lengths of the lncRNAs to that of the RefSeq genes (both protein-coding and non-coding). As a result, the novel lncRNAs represent shorter putative ORF length than either RefSeq protein-coding RNAs (∼0.17 kb vs ∼1.6 kb, *P *< 2*.*2 × 10^−16^) or ncRNAs (∼0.17 kb vs ∼0.30 kb, *P *< 2*.*2 × 10^−16^), consistent with a previous report on zebrafish embryonic lncRNAs [16]. Although the novel lncRNAs candidates are to be ncRNAs, they can differ from the RefSeq ncRNAs used for comparison in some features due to several reasons as follows. First, the RefSeq ncRNAs do not only include lncRNAs, but also other categories of ncRNAs, e.g. microRNAs and small nucleolar RNAs. Second, the lncRNAs can be further classified according to their biological functions, thus the features of different categories of lncRNAs may differ from each other. The lncRNAs we detected may not come from the same category as that annotated by RefSeq. Third, the unbalanced population sizes can affect the comparison between the two categories of ncRNAs. Last, the putative ORF length of the lncRNAs we predicted were limited (< 300 nt), which can affect the ORF comparison. Therefore it is reasonable to see that the two categories of ncRNAs represent slight statistical difference, which is far less than that between the mRNAs and ncRNAs.

### Novel lincRNAs have lower expression level than protein-coding transcripts

**Figure 8:**
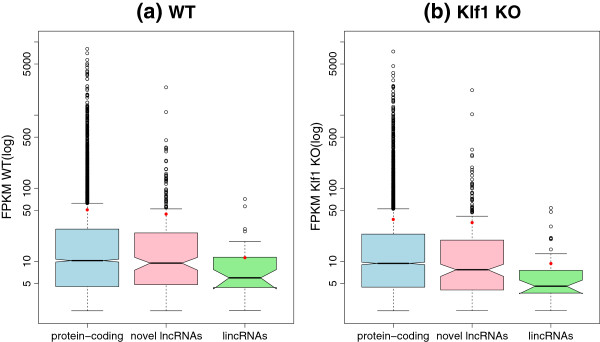
**Comparison of expression level between protein-coding transcripts and novel lncRNAs. (a)** In the WT condition, the protein-coding transcripts (∼50.92) represent slightly higher expression level than the novel lncRNAs (∼44.54), but significantly higher expression than the lincRNAs (∼11.29) extracted from the lncRNAs; **(b)** In the *Klf1* KO condition, the protein-coding transcripts (∼37.63) also show slightly higher expression level than the lncRNAs (∼34.06), but significantly higher expression than the lincRNAs (∼9.6). In addition, the protein-coding transcripts and the novel lncRNAs represent similar median expression in either WT (10.29 vs 9.509) or *Klf1* KO (9.421 vs 7.722) condition. All means are marked by red points

Previous studies also showed that lncRNAs are expressed at significantly lower levels than are protein-coding transcripts [13,14,16]. To determine whether the embryonic lncRNAs we detected have the same expression feature, we compared the quantified expression levels (FPKM) of the 308 novel lncRNAs to that of the known protein-coding transcripts (Figure 8). In the WT condition (Figure 8-a), the protein-coding transcripts represents slightly higher expression than the novel lncRNAs on average (∼50.92 vs ∼44.54, *P *= 0*.*554, Welch Two Sample t-test). Similarly, in the *Klf1* KO condition (Figure 8-b), the protein-coding transcripts also show slightly higher expression than the lncRNAs on average (∼37.63 vs ∼34.06, *P *= 0*.*6986). The comparison result indicates that the total novel lncRNAs do not show significant lower expression than the protein-coding ones. Moreover, we extracted the 52 lincRNAs (‘u’ classcode) from the 308 lncRNAs for the expression comparison. The result manifests that the lincRNAs we predicted represents significant lower expression than the protein-coding ones in either WT or *Klf1* KO condition (∼11.29 vs ∼50.93, *P *< 2*.*2 × 10^−16^, and ∼9.38 vs ∼37.63, *P *< 2*.*2 × 10^−16^, respectively).

### Differentially expressed lncRNAs

**Figure 9:**
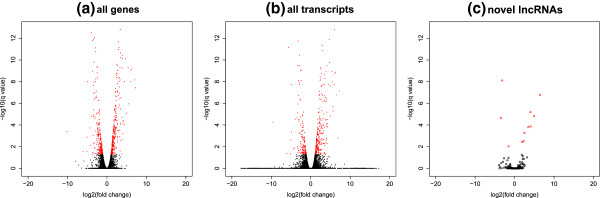
**Differential expression of transcripts between WT and*Klf1* KO.** The three volcano plots illustrate the differential expression (DE) between the WT and *Klf1* KO samples at either gene or transcript level: **(a)** DE of all genes. At the gene level, *Klf1* globally appears to be an activator since more genes are significantly repressed (334, red points over the positive x-axis) than the activated ones (250, red points over the negative x-axis) after *Klf1* is knocked out; **(b)** DE of all transcripts. At the transcript/isoform level, *Klf1* also behaves like an activator since more transcripts are significantly repressed (262) than activated ones (147) after *Klf1* is knocked out; **(c)** DE of the novel lncRNAs. For the 13 DE significant lncRNA transcripts, *Klf1* still functions like an activator since 10 lncRNAs are repressed and 3 ones are activated after *Klf1* is knocked out. The DE significant transcripts are all represented by red points

Using cuffdiff, we conducted the differential expression (DE) tests between the WT and *Klf1* KO samples for analysing the function of the novel lncRNAs. At the gene level (Figure 9-a), *Klf1* represents like an activator since more assembled genes are significantly repressed (334) after *Klf1* is knocked out than the activated ones (250). At the transcript level (Figure 9-b), *Klf1* also behaves like an activator since more transcripts are significantly repressed (262) after *Klf1* is knocked out than the activated ones (147). Moreover, we detected 13 (Additional file 4) novel lncRNAs with DE significant. Notably, *Klf1* still functions like an activator for the 13 lncRNAs (10 repressed vs 3 activated after *Klf1* is knocked out, Figure 9-c). Thus it is obvious that *Klf1* can function as an activator globally, regulating the expression of a number of genes or transcripts including the lncRNAs we detected. The detailed categories of the 13 lncRNAs of DE significant can be seen from Table 5.

**Table 5:** Categories of novel lncRNAs of differential expression significant

**Class code**	**Transcript number**	**Percentage**	**Description**
j	5	38.46%	At least one splice junction is shared with a reference transcript
i	1	7.69%	A transfrag falling entirely within a reference intron
o	2	15.38%	Generic exonic overlap with a reference transcript
u	4	30.77%	Unknown, intergenic transcript
x	1	7.69%	Exonic overlap with reference on the opposite strand
total	13	100%	Total

However, cuffdiff does a length correction that has a tendency to inflate the FPKM counts for small transcripts, which can interfere the differential expression analysis. To alleviate this problem, we re-ran the DE tests with the “–no-effective-length-correction”parameter. As a result, we obtained the same results as that without the parameter, which represent the robustness of our predictions.

## Discussion

RNA-Seq has been revolutionizing the transcriptome study as it can effectively capture the whole transcriptome of various cell types under different conditions. Here we predicted 308 novel mouse embryonic lncRNAs from the RNA-Seq data of WT and *Klf1* KO samples using a computational pipeline. The novel lncRNAs we detected represent shorter transcript length, fewer exons and shorter putative ORF length, and the 52 lincRNAs of the lncRNAs show lower expression level, compared with known protein-coding transcripts. Moreover, we identified 13 differentially expressed novel lncRNAs, which may be regulated by *Klf1* and play functional roles in the development of erythroid cells potentially. Notably, two lncRNAs (IDs: 2_00016377 and 2_00016378) we predicted represent almost the same structures as another two lncRNAs predicted by Tallack et al. [27] based on the same dataset. Specifically, most exons of 2_00016377 and 2_00016378 match that of their ‘lincred1-giant’ and ‘lincred1-dwarf’ lncRNAs respectively. The slight difference may be caused by both of the strategies of transcriptome reconstruction and program versions used. Despite of that, the differential expression of the two lncRNAs we detected can be explained by Tallack et al.’s validation using **R**eal-**t**ime **Q**uantitative **PCR** (qRT-PCR) [49] on their ‘lincred1’ lncRNAs.

On the other hand, our pipeline followed a similar strategy for predicting human lincRNAs [13], but we differ in three aspects. First, we used FPKM as a feature for filtering low quality assemblies instead of the read coverage [13] due to the fact that FPKM can unbiasedly represent the expression level of a transcript and the read coverage does not show better performance than FPKM in classifying the complete and partial transcripts assembled from our data (AUCs are equal). Second, we excluded the transcripts having long putative ORF length (≥300 nt), which was previously used by the FANTOM consortium [50]. This arbitrary cutoff makes our predictions more stringent, but it must omit the lncRNAs having long putative ORF (≥300 nt). Last, we detected several DE significant lncRNAs, which composed a subset of the total lncRNAs we detected and they are more worth being investigated by loss and gain of function studies than the other novel lncRNAs in our scenario. Consequently, our computational methods can effectively alleviate further experimental work for studying the lncRNAs that may participate in the development of erythroid cells.

Although our method presented its ability in detecting novel lncRNA candidates, its prediction accuracy can be improved from several aspects, such as using more reliable reads generated by high-quality deep sequencing, paired-end sequencing and strand-specific sequencing. And recent single-molecule sequencing technologies can provide more unbiased ways to capture the transcriptome [51]. The sensitivity of transcriptome reconstruction can also be improved by using various strategies, such as integrating assembly results from Scripture [14]. In addition, the novel lncRNAs predicted from our computational pipeline should be validated by biological experiments, such as cloning and PCR-based techniques [22] as several ones have been tested in the original study by Tallack et al. [27]. Furthermore, additional genetic and/or epigenetic data sources, e.g. **C**hromatin **I**mmunoprecipitation-**Seq**uencing (ChIP-Seq) on chromatin signatures, can be valuable sources providing useful information for characterizing functions of the novel lncRNAs. And the loss and gain of function studies can be conducted for exploring regulatory mechanisms of the lncRNAs.

## Conclusions

We predicted a set of novel lncRNAs using our computational pipeline from the RNA-Seq data of *Klf1* knockout study, and the DE significant lncRNAs are worth being further studied with regard to their biological functions.

## Competing interests

The authors declare that they have no competing interests.

## Authors’ contributions

LS designed and implemented the method. ACP and MRT provided the raw datasets. LS carried out the experiments and analysis. LS, ZZ, TLB, ZX and HL wrote the paper. All authors read and approved the final manuscript.

## Supplementary Material

Additional file 1 **Supplementary materials.** It contains supplementary materials supporting the main text. Click here for file

Additional file 2 **GTF of high-quality assembled transcripts.** It is a GTF file recording the 28963 high-quality assemblies, which were used in the differential expression tests. And their structures can be visualized by UCSC genome browser. Click here for file

Additional file 3 **GTF of novel lncRNAs.** It is a GTF file recording the 308 novel lncRNAts detected by lncRScan. The transcript structures can be visualized by UCSC genome browser. Click here for file

Additional file 4 **GTF of differentially expressed lncRNAs.** It is a GTF file recording the 13 differentially expressed lncRNAs between the WT and *Klf1* KO samples. The transcript structures can be visualized by UCSC genome browser. Click here for file
