# Supplementary material for: Prediction of novel long non-coding RNAs based on RNA-Seq data of mouse Klf1 knockout study
Source: BMC Bioinformatics. 2012 Dec 13;13:331. doi: 10.1186/1471-2105-13-331 (PMC3577497; doi:10.1186/1471-2105-13-331)
Supplement: Additional file 1 — Supplementary materials. It contains supplementary materials supporting the main text. [file 1471-2105-13-331-S1.ZIP › Supplementary_materials.doc]

**Supplementary materials**

1. **Analyze the reproducibility of read mapping**

We defined the overlap ratio (OR) to compare the read coverage across replicates in the same condition by a formula below.

$$OR=\frac{\sum_{i} m_{i}}{\sum_{i} m_{i}+\sum_{j} n_{j}}$$

Where *m_i_* represents the length of an interval overlaps that of any other replicate in the same condition. And *n_j_* denotes the length of an interval without overlap.

Supplementary Table 1: Overlap ratios in Klf1 KO and WT conditions

| Condition | Overlap ratio |
| --- | --- |
| Klf1 KO | 0.4782 |
| WT | 0.4218 |

The ORs above show that the read coverage over the genome does not represent good consistent across replicates due to both of the low sequencing depth and variability across the replicates. However, the gene expression estimated from the mapped reads of the replicates represented consistent with each other (Supplementary Figure 1, Tables 2 and 3). Similarly, the isoform expression estimated from the replicates also represented consistent with each other (Supplementary Figure 2, Tables 4 and 5).

|  |  |
| --- | --- |
|  |  |
|  |  |
| Supplementary Figure 1: The three figures (left) represent the gene expression comparison between replicates of the Klf1 KO condition while the other three ones (right) show the comparison between replicates of the WT condition. | |

Supplementary Table 2: Correlation coefficients

of gene expression between replicates of Klf1 KO

|  | KO_1 | KO_2 |
| --- | --- | --- |
| KO_2 | 0.966 |  |
| KO_3 | 0.845 | 0.857 |

Supplementary Table 3: Correlation coefficients

of gene expression between replicates of WT

|  | WT_1 | WT_2 |
| --- | --- | --- |
| WT _2 | 0.950 |  |
| WT _3 | 0.942 | 0.886 |

|  |  |
| --- | --- |
|  |  |
|  |  |
| Supplementary Figure 2: The three figures (left) represent the isoform expression comparison between replicates of the Klf1 KO condition while the other three ones (right) show the comparison between the replicates of the WT condition. | |

Supplementary Table 4: Correlation coefficients

of isoform expression between replicates of Klf1 KO

|  | KO_1 | KO_2 |
| --- | --- | --- |
| KO_2 | 0.966 |  |
| KO_3 | 0.844 | 0.853 |

Supplementary Table 5: Correlation coefficients

of isoform expression between replicates of WT

|  | WT_1 | WT_2 |
| --- | --- | --- |
| WT _2 | 0.943 |  |
| WT _3 | 0.937 | 0.871 |

2. **An ID list of lncRNAs that have the same structure as NONCODE 3.0 annotation**

Supplementary Table 6: An ID list of 5 identical lncRNAs

| Transcript ID | NONCODE ID | Genbank Accession |
| --- | --- | --- |
| 2_00006252 | n278144 | [AK163398](http://www.ncbi.nlm.nih.gov/nucleotide/AK163398) |
| 2_00006749 | n294621 | [AK013406](http://www.ncbi.nlm.nih.gov/nucleotide/AK013406) |
| 2_00006887 | n344232 | N/A |
| 2_00009769 | n279007 | [AK043703](http://www.ncbi.nlm.nih.gov/nucleotide/AK043703) |
| 2_00018871 | n289698 | [AK031724](http://www.ncbi.nlm.nih.gov/nucleotide/AK031724) |
